# Bioethics as *bios ethikos*

**DOI:** 10.1007/s11019-026-10367-x

**Published:** 2026-05-30

**Authors:** Luis de Miranda

**Affiliations:** 1 Centre for Research Ethics and Bioethics, Uppsala University, Box 564, Uppsala, 751 22 Sweden; 2 Turku Institute for Advanced Studies, University of Turku, Turku, Finland

**Keywords:** Bioethics, Bios ethikos, Existential remainder, Philosophical health, 4E Cognition

## Abstract

Drawing on a genealogical analysis of the distinction between *zoē* (biological life as organic functioning) and *bios* (a distinctively human way of life shaped by meaning, orientation, and evaluative practice), the article reconceives bioethics as *bios ethikos*: ethical reflection on the conditions under which forms of life become meaningful and inhabitable. It introduces the notion of the existential remainder to describe the ethically significant dimensions that persist when institutional deliberation leaves aspects of lived existence under-articulated. The article proposes a renewed structural orientation grounded in the heuristic formula T = PEWS + C. Ethical thinking (T) is distributed across four interrelated domains of lived existence: Person, Earth, Work, and Society (PEWS), while the addition of C designates the creative openness that resists full institutional codification. PEWS-oriented evaluation may be operationalised through more integrative assessment tools, while maintaining vigilance toward the irreducible horizon of existential creativity. Bioethics, thus reconceived, becomes not only the regulation of life, but reflection on whether the governance of life sustains the conditions under which life can remain meaningfully lived.

## Introduction

Bioethics is among the most pervasive yet conceptually indeterminate terms in contemporary ethics. Invoked across domains ranging from intensive care and genomics to public health and algorithmic medicine, it is commonly framed as applied ethics: a regulatory discipline designed to adjudicate competing claims, secure consent, prevent harm, and guide biomedical practice (Beauchamp and Childress 2019). Bioethics provides indispensable procedural legitimacy, yet the institutional consolidation of the discipline also brings into view a recurring phenomenon: certain ethically defensible decisions appear to leave dimensions of lived experience insufficiently articulated. I refer to these unresolved dimensions as *existential remainder*.

To make sense of this boundary, the article returns to an older distinction that the modern term bioethics tends to flatten: the distinction between *zoē* (biological life as somatic functioning) and *bios* (a distinctive way of life shaped by meaning, orientation, and evaluative practice). Bioethics, by its very etymology, can be understood as centrally concerned with βίος ἠθικός (*bios ethikos*): a process of ethical reflection on forms of life as lived and valued. When ancient Greek philosophers, chief among them Aristotle, qualified *bios* with adjectives (βίος θεωρητικός, the theoretical way of life; βίος πολιτικός, the political way of life; βίος ἀπολαυστικός, the pleasurable way of life), they indicated that ethics addresses manners of existing and evaluating: which *bios*, and which form of existence can be called good or flourishing (Aristotle 1999; NE I.5, 1095b17–22).

The purpose of this article is to clarify how the institutional history of bioethics has increasingly centred on zoē (somatic viability) while leaving *bios ethikos* insufficiently articulated as an object of collective ethical reflection. By tracing how the question of which life to live was transformed and by diagnosing how institutional bioethics can generate residual ethical significance at its own boundaries, I argue in what follows that bioethics can be reconceived as the articulation of conditions under which lives worth living become possible and compossible (what is possible together).

This article proceeds in four steps. It first sketches a genealogy from ancient philosophy to modern biomedicine to show how the question of which life to live was displaced by the imperative to preserve life as such. It then analyses how this inheritance shapes institutional bioethics and reveals or generates existential dimensions. Third, it introduces eudynamic bioethics as a structural orientation across Person, Earth, Work, and Society (PEWS). Finally, it outlines some implications for clinical practice, institutional reflection, and bioethical education.

## The displacement of *Bios*: a genealogical account

To understand why contemporary bioethics tends to centre on somatic viability and procedural legitimacy, it is necessary to revisit a distinction that has played a recurring, though contested, role in the philosophical understanding of life. In ancient Greek thought, *bios* did not denote life in general, but a qualifiable manner of living, a form of existence shaped by orientation, practice, and evaluative commitment. Ethics, in this context, was not primarily concerned with preserving life as such, but with the question of which bios could be called good, admirable, or philosophically healthy.

This is most clearly articulated in Aristotle’s ethical writings. In the *Nicomachean Ethics*, Aristotle distinguishes between different *bioi* – *bios theoretikos*, *bios politikos*, and *bios apolaustikos* – each corresponding to a distinct way of inhabiting the world and relating to value (NE I.5, 1095b17-22). As commentators have long noted (e.g. Ackrill 1980; Irwin 1999), what is philosophically significant here is the implication that bios is intrinsically normative: a life is ethically intelligible only insofar as it is oriented, evaluable, and shared within practices.

This conception of ethics as reflection on ways of life was not confined to Aristotelian philosophy. As Pierre Hadot has argued, ancient philosophy more broadly functioned as a set of spiritual and practical exercises aimed at transforming how one lives, perceives, and evaluates existence (Hadot 1995). To adhere to a philosophical school was to inhabit a distinctive bios, characterised by particular relations to embodiment, finitude, community, and the cosmos. Ethics, in this sense, was inseparable from lived orientation.

The prominence of *bios* as an ethical category was progressively transformed with the emergence of Christian theology. Early Christian thought reconfigured the Greek distinction between *bios* and *zoē*, elevating *zoē* to signify eternal life and participation in divine being, while casting earthly *bios* in more ambivalent terms associated with transience, temptation, and moral vulnerability (Arendt 1958; Agamben 2016). Ethical attention shifted accordingly, from the cultivation of admirable forms of life in this world to preparation for salvation and eternal life beyond it.

Modernity did not abandon this transformation so much as translate it. As Christian theological categories were secularised, life increasingly became an object of scientific description and technical intervention (Foucault 1973). With the rise of anatomy, physiology, and later biomedicine, *zoē* understood as organic functioning became the primary bearer of value. Ethical concern was increasingly articulated in terms of preserving, extending, and optimising life in general, often independently of questions concerning the qualitative orientation of existence.

This transformation was not merely institutional or technological, but also semantic. In early modern scientific discourse, the term *life* increasingly came to designate measurable, observable, and manipulable processes (circulation, respiration, metabolism, reproduction, etc.) rather than a lived form of existence. As a result, the prefix *bio-* was progressively reinterpreted through the lens of the life sciences. What had once named a qualitative mode of existence was redefined to denote somatic processes, what medicine and science call *biological*. Ethical attention thus came to be oriented toward the preservation and optimisation of life understood in physiological terms, while the ancient question of *bios*, of how life is lived, oriented, and evaluated, was increasingly displaced from the centre of health-related reflection.

It is against this background that modern bioethics takes shape. Emerging in the second half of the twentieth century, bioethics presents itself as a critical response to the expanding power of biomedicine: a domain of reflection intended to humanise medical practice, protect vulnerable subjects, and articulate ethical limits to technical intervention. In doing so, bioethics introduces indispensable principles (respect for autonomous persons, non-maleficence, justice, informed consent, etc.) that attempt to correct the excesses of a purely technical or scientific rationality. 

Still, this corrective gesture also carries an implicit presupposition. In addressing the ethical limits of biomedical intervention, bioethics in general (although not all branches of it), and principlism in particular, largely accepts the object of biomedicine itself: life in general understood as biological or somatic functioning being the primary locus of ethical concern. Ethical reflection is thus all too often oriented toward how life in general should be protected, distributed, or regulated, rather than toward reopening the more fundamental question of which forms of life are being sustained, enabled, or foreclosed. In this sense, many forms of contemporary bioethics operate within the biomedical framing of life and contribute to its normative stabilisation.

One consequence of this inheritance is a subtle but far-reaching shift in the ethical axis itself. Where much of classical ethical reflection was organised around the distinction between a good life and a deficient or distorted way of living, modern biomedical and public-health rationalities increasingly organise ethical concern around the opposition between life and death. Death comes to function as the ultimate negative value to be postponed, minimised, or managed, while life is implicitly treated as a self-evident good insofar as it persists. Ethical success is thus often measured in terms of survival rates, risk reduction, and mortality prevention, rather than in relation to the qualitative articulation of existence, vitality, orientation, creativity, purpose, or the capacity to inhabit a meaningful form of life.

This is not to deny the importance of preventing premature death, but it identifies a shift in orientation: when avoiding death becomes the dominant horizon, ethics tends to be organised around survival metrics rather than the qualitative articulation of existence. What becomes central is mortality management; what recedes is a reflection on how life is meaningfully lived. Giorgio Agamben, influenced by Foucault’s biopolitics, has argued that modern ethical and political rationalities increasingly centre on organic life (*zoē*), while forms of life (*bioi*) recede from explicit consideration (Agamben 1998, 2016). Agamben’s genealogy is influential but contested, and its historical scope and political conclusions have been subject to critical debate (Fassin 2009; Lemke 2011; Mills 2018). Nevertheless, his analysis remains analytically useful insofar as it helps to name a broader tendency: the displacement of *bios* as an explicit object of ethical reflection, even within practices, such as bioethics, that aim to humanise biomedical power.

This genealogy does not suggest a mere pragmatic failure of bioethics, nor does it deny the presence of rich philosophical reflection within the field. Rather, it clarifies a structural inheritance: an ethics originally concerned with ways of living has been progressively re-centred on generic life and survival. As a result, contemporary bioethics can succeed in regulating biomedical practice while nevertheless encountering situations in which something humanly significant remains unresolved, situations in which questions of how to live, what kind of life is being sustained, or which forms of existence are being enabled or foreclosed reappear at the boundaries of established ethical categories.

## Existential remainder and institutional rationality

If the preceding analysis has shown how contemporary bioethics inherits a focus on life understood primarily as somatic viability, the question that follows is not only whether this orientation is ethically misguided, but what it does when translated into institutional practice. Across clinical, research, and public health contexts, bioethics is rarely encountered as abstract moral theory. It is enacted through committees, review procedures, protocols, and governance structures tasked with guiding action under conditions of uncertainty, constraint, and accountability. In such settings, ethical deliberation must be rendered actionable, publicly defensible, and procedurally legitimate. Decisions must be justifiable according to shared criteria; reasons must be recordable, contestable, and transferable across cases. This procedural rationality is indispensable. Without it, ethical decision-making would lack consistency, fairness, and institutional authority (Jonsen 1998; Beauchamp and Childress 2019).

It is precisely this success that gives rise to a recurring and often unsettling phenomenon. Ethically justified decisions, decisions that satisfy procedural requirements, that align with established principles, and that withstand institutional scrutiny, are frequently accompanied by a sense that something important has not been fully addressed. This sense does not arise because ethical reasoning has failed, nor because decision-makers have acted negligently or without care. Rather, it emerges when ethical reasoning succeeds within a framework that is not designed to articulate certain dimensions of lived existence. What remains unresolved in such cases is not a disagreement about principles or a lack of information, but a residue of ethical significance that resists procedural articulation.

We may refer to these residues as the *existential remainder*. Existential residues are not subjective afterthoughts, psychological burdens, or signs of moral weakness. Nor are they reducible to inefficiencies or gaps in ethical reasoning. They name a recurring structural feature of institutional bioethical settings: the tendency for ethically significant aspects of *bios* (ways of living, forms of orientation, questions of meaning, identity, and greater purpose) to appear at the margins when ethical deliberation is organised primarily around the regulation of somatic viability, risk and mortality management. The existential remainder is thus not an anomaly to be eliminated, but a predictable by-product of an ethical rationality shaped by the life-death opposition.

A brief illustration may help. As a result of a routine end-of-life decision in an intensive care unit, life-sustaining treatment is withdrawn from a patient with no realistic prospect of recovery, in accordance with the patient’s prior wishes, family agreement, and unanimous clinical judgment. The decision is procedurally faultless. Yet in the days that follow, several team members report a persistent unease that they cannot fully articulate, the family describes a difficulty in narrating what happened, and the unit’s collective sense of meaningful care is briefly destabilised. Nothing has gone wrong by any institutional measure; nevertheless, something ethically significant has remained without expressive form. This is an example of the existential remainder in its ordinary clinical texture.

The philosophical significance of this claim becomes clearer when placed in dialogue with Bernard Williams’s influential critique of ethical theory (Williams 1985). Williams famously argued that even when an agent acts for the best available moral reasons, something of ethical importance may be lost that cannot be absorbed into the justification of the action itself. In situations of tragic conflict, ethical reasoning may tell us what ought to be done, but it cannot fully reconcile us to what has been sacrificed in doing it. Regret, loss, or a sense of moral damage may persist without implying that the action was wrong. For Williams, this persistence reveals the limits of moral theory: ethical life cannot be fully captured by abstract principles or decision procedures without residual existential significance.

Williams’s analysis, however, is primarily articulated at the level of individual moral agency. What the present argument suggests is that a structurally analogous phenomenon occurs at the level of institutions. When ethical deliberation is formalised through procedures designed to secure legitimacy, consistency, and accountability, the losses and dislocations generated by ethically justified decisions are often displaced rather than resolved. The existential remainder conceived within *bios ethikos* thus extends Williams’s insight beyond individual moral psychology to institutional ethical rationality. It names the condition in which ethically significant dimensions of lived existence exceed the expressive capacity of established ethical frameworks, because they are oriented toward a different object.

This institutional displacement helps to clarify the status of phenomena commonly described as moral distress. Moral distress has been widely documented among healthcare professionals and is often interpreted as an individual psychological response to being constrained from acting on one’s moral judgment (Jameton 1984; Epstein and Hamric 2009). Without denying the affective reality of such experiences, the present analysis suggests that moral distress can also be understood as an empirical signal of an existential dimension. What is experienced as distress may arise when individuals encounter the limits of institutional ethical vocabularies, when they are required to act in ways that are ethically justified within procedural frameworks, yet remain difficult to integrate into a coherent sense of a life well lived or a practice worth inhabiting. In such cases, distress does not diagnose ethical error so much as it reveals a mismatch between what institutions are equipped to govern and what forms of existence continue to demand ethical attention.

Importantly, recognising the existential and philosophical dimensions does not entail that every ethical decision must resolve it, nor that institutional actors are responsible for addressing all dimensions of human existence in every context. Ethical responsibility is necessarily distributed across roles, levels, and temporal horizons. Clinical teams, ethics committees, administrators, and policymakers operate under different constraints and with different capacities for action. To acknowledge the existential remainder is to clarify where ethical significance is being produced, displaced, or externalised. It invites reflection on how institutional arrangements shape what can appear as ethically relevant, and on how questions concerning *bios*, forms of life as lived and meaningfully valued, are deferred, delegated, or rendered invisible within ethical governance.

It is equally important to acknowledge that existential remainders rarely arise in pure form. In the practical reality of ethics committees and clinical units, what presents itself as residual ethical significance is often entangled with, and sometimes produced by, more pragmatic constraints: scarcity of resources, time pressure, rigid institutional hierarchies, and competing administrative demands (Pellegrino 1992; Jonsen 1998). Recognising the existential remainder does not require romanticising it. The distinction that matters is rather between the *conditions* that produce remainders, which frequently include such pragmatic factors, and the *nature* of what remains, which is irreducibly existential and philosophical rather than merely logistical. A more adequate diagnosis of remainders therefore reads them as situated phenomena: simultaneously shaped by material constraints and irreducible to them. In many institutional settings, indeed, scarcity and structural pressure do not merely accompany existential remainders but actively generate them, by foreclosing the time, attention, and relational space within which questions of meaning could be addressed in the first place.

Seen in this light, existential and philosophical residues become a condition to be recognised and interpreted. This may open the boundary at which institutional bioethics reaches the limits of a principle-based rationality. At that boundary, the question is no longer simply how to decide correctly within existing frameworks, but how to articulate the conditions under which lives remain meaningfully inhabitable, and capable of sustaining a civilizational purpose beyond survival alone. In this respect, the existential remainder can be understood as a boundary phenomenon, in a sense resonant with Karl Jaspers’s notion of *Grenzsituationen*: not a problem to be resolved by improved technique, but a limit at which the conditions and meaning of existence come into view (Jaspers 1959). It is here, at the point where procedural ethics succeeds yet something humanly significant remains at stake, that a reorientation toward *bios ethikos* becomes both necessary and intelligible.

## Eudynamic bioethics as a heuristic for *bios ethikos*

If the existential remainder reveals the limits of what institutional bioethics can legitimately articulate, the preliminary response is not to dissolve or expand that boundary, but to clarify its structure. What is required at first is not a new normative doctrine that would dictate substantive values, nor an expansion of institutional responsibility into domains it cannot practically assume. Rather, what is needed is a way of making intelligible how ethical significance arises across the conditions that shape lived human existence, and how institutional rationalities distribute attention across those conditions. It is in this diagnostic and orientational sense that the present article proposes a *eudynamic* orientation in bioethics.

The concept of *eudynamia* that grounds this orientation requires a brief clarification. As first hypothesised in earlier work on philosophical health (de Miranda 2021), eudynamia does not denote vitality, strength, or wellbeing in a physiological or psychological sense, but names a normative relation to *dynamis* understood as potentiality: the capacity to access, actualize, compose, and sustain meaningful and compatible possibilities over time. Drawing on the Greek term *dynamis* (signifying potential, possible), eudynamia designates a process of *well-relatedness to possibility*.

In this sense, eudynamia differs both from hedonic accounts of wellbeing and from classical eudaimonic models centred on the realization of a predefined human telos. It does not specify what a good life should be, but asks whether persons, practices, and institutions are enabled within conditions of possibility and compossibility in ways that are generative rather than foreclosing. Eudynamia thus functions as an orientational concept: it shifts ethical attention from outcome optimization or norm compliance toward the cultivation and preservation of capacities for meaningful becoming, individually and collectively. Within bioethical contexts, this entails asking not only whether decisions preserve life or satisfy procedural criteria, but whether they sustain the conditions under which lives can continue to take form as intelligible, inhabitable, composable, and worth living.

*Eudynamic bioethics* offers a structural orientation for understanding how ethical meaning is generated, displaced, or deferred across the lived conditions of human life once somatic survival is secured. It also draws on developments in cognitive science associated with what has been called the 4E paradigm (embodied, embedded, enactive, and extended cognition) while also acknowledging the limits and internal fragmentation of that paradigm.

Since the early 1990s, 4E approaches have decisively challenged computational and representational models of mind by emphasizing that cognition is not confined to internal information processing but is distributed across bodies, environments, social or skilled practices, and material scaffolds (Varela, Thompson, and Rosch 1991; Clark and Chalmers 1998; Thompson 2007; Gallagher and Zahavi 2008). Embodied cognition highlights the constitutive role of the lived body and its outer or inner perceptions, including emotions; embedded cognition stresses the dependence of cognition on sociocultural contexts; enactivism emphasizes sense-making as an activity enacted through dexterous interaction; and extended cognition argues that cognitive processes may include tools, technologies, and ecological spaces beyond the skin and skull (Robbins and Aydede 2009; Shapiro 2014; Gallagher 2021).

Human cognition and sense-making valuation unfold as lived experience structured by horizons of possibility and compossibility. Experience is not neutral observation but an oriented engagement with what can be done, sustained, and lived in interconnection. This insight, developed in phenomenology from Husserl to Merleau-Ponty, allows ethical reflection to be reframed as attention to how possibilities are opened, constrained, and composed into coherent forms of life (Husserl 1936/1970; Merleau-Ponty 1945/2012; Thompson 2007; Gallagher 2013). In more pragmatic terms, this means that every clinical situation is encountered not as a neutral list of options but as a felt sense of what is open, foreclosed, or worth pursuing within a given form of life or biography, and that ethical attention should be sensitive to this horizon.

On this basis, I propose the PEWS domains of eudynamia – an acronym for Person, Earth, Work, and Society – as an interpretive translation of the four dimensions along which 4E cognition distributes lived significance. These dimensions correspond to the embodied, extended, enactive, and embedded aspects of experience, translated into existentially and ethically legible terms. Person names *embodied* experience: relational affect, identity, biographical sense-making and the lived sense of meaningful orientation. Earth refers to the material, technological, and ecological scaffolding that *extends* cognition and sustains life beyond the individual. Work designates the *enactive* dimension: practices, roles, responsibilities and activities through which meaning emerges from acting and world-making. Society captures embeddedness in social norms, institutions, cultures, languages, histories, and collective expectations.

Within this scheme, it is worth noting that *Earth* merits a more substantive articulation than the standard 4E literature on extended cognition tends to provide. Earth in eudynamic bioethics designates not only material and technological scaffolding (which are indeed important), but also the planetary and ecological horizon within which a form of life is situated. Earth is thus where ethical reflection encounters the limits of the strictly human and opens onto questions of ecological belonging, planetary finitude, and the conditions under which forms of life remain inhabitable beyond anthropocentrism.

The PEWS dimensions are analytically distinct but practically inseparable. Ethical situations do not arise within one dimension alone; they emerge from their interaction. PEWS makes visible how institutional bioethical reasoning tends to foreground certain dimensions, most often somatic viability at the level of the person and procedural legitimacy at the level of society, while leaving other aspects under-articulated or externalised as remainder. In dealing for example with a patient receiving a diagnosis of multiple sclerosis, basic ethical attention focuses on informed consent and treatment choice. A PEWS-informed reading would distribute the ethical question integratively. At the level of *Person*, the diagnosis disrupts embodied self-experience and biographical continuity; at the level of *Earth*, it changes the patient’s relation to the technological and ecological infrastructures of treatment, mobility, and care; at the level of *Work*, it reconfigures vocational identity, capacity, and the meaning of daily activity; at the level of *Society*, it shifts the patient’s identity within networks of recognition and dependence.

This is where the notion of existential remainder returns with greater precision. Boundary phenomena arise when ethical deliberation succeeds according to institutional criteria (risk mitigation, compliance, allocation, etc.), yet leaves unresolved questions concerning orientation, meaning, integrity, moral distress or collective direction. From a PEWS perspective, such dimensions are not anomalies but structural by-products or conditions for how ethical attention is distributed. A clinical decision may be justified with respect to somatic outcomes while leaving the enactive dimension of professional work ethically strained, or the societal narratives of worth implicitly reinforced without reflection. A policy may optimise population-level outcomes while externalising existential costs to individuals or communities.

Eudynamic bioethics does not treat these remainders as failures to be eliminated. Nor does it interpret them as purely subjective residues beyond ethical concern. Instead, it understands them as boundary phenomena, signals that ethical significance has exceeded the expressive capacity of a given institutional frame. These limit situations and boundary concepts may generate experiences of guilt, suffering, powerlessness or finitude (Jaspers 1913/1997; Plessner 1928/2019).

From this perspective, the task of bioethics is to clarify which dimensions are being actively governed, which are deferred, and which are rendered invisible. Eudynamic bioethics thus operates as an ethics of articulation; it illuminates how ethical decisions shape the conditions under which lives can be lived, sustained, and made sense of over time within the arrangement of Person-Earth-Work-Society (PEWS).

Recognising the relevance of Earth or Society does not necessarily imply that frontline clinicians, for instance, must always take responsibility for planetary health or social transformation. Rather, it allows ethical reflection to distinguish between levels of responsibility and timescales of action. Some dimensions may be appropriately addressed through immediate clinical judgment; others may require institutional reflection, policy revision, or cultural change. What matters chronologically is not that all dimensions be resolved systemically, but first and foremost that their ethical significance be acknowledged and discussed rather than silently displaced.

In this way, eudynamic bioethics complements existing approaches in clinical ethics, organizational ethics, and public health ethics. Principles such as autonomy, beneficence, non-maleficence, and justice remain useful tools. What eudynamic bioethics adds is a way of situating these tools within a broader horizon of *bios ethikos*: ethical reflection on forms of life as lived meaningfully and valued across PEWS. By grounding this reflection in a phenomenologically informed reading of 4E cognition, of which more below, the approach provides a disciplined, non-utopian framework for understanding how ethical significance emerges at the boundary between institutional rationality and lived human existence.

## Philosophical health as framework for bioethics

The six dimensions developed in what follows are not a second, parallel framework competing with PEWS, but the phenomenological articulation of how sense-making is distributed across the four PEWS domains. PEWS designates the cognitive-distributional architecture of lived existence, drawn from a reading of 4E cognition theory; the six elements of philosophical health designate the experiential textures through which this architecture is concretely inhabited as *bios ethikos*. Each dimension has a primary anchor in one or two of the PEWS domains, while extending into others, since lived experience exceeds analytical boundaries.

If bioethics is to articulate more fully the ethical dimensions of *bios*, it requires a framework capable of articulating multiple dimensions of flourishing without prescribing a single vision of the good life. Philosophical Health names such a framework, complementary to physical and psychological health: a structural account of sense-making across six interrelated dimensions – bodily sense, sense of self, belonging, possibility, purpose, and philosophical sense – understood as mutually reinforcing aspects of a compossible and meaningful life (de Miranda 2024).

In what follows, I briefly elaborate each dimension and indicate how it might reframe bioethical practice. This is not a prescriptive account of *what* people should value, but a structural account of *how* humans make sense of their lives and what conditions enable such sense-making to flourish eudynamically. The framework does not prescribe what content these dimensions should have; it describes structural conditions that are transcultural: humans across traditions make sense through embodiment, narrative, belonging, imagination, purpose, and ideology, philosophy or cosmology.

*Bodily Sense*: At the most immediate level, human life is embodied. Bodily sense is anchored primarily in the Person dimension of PEWS, with extensions into Earth (the body as ecological interface) and Work (skilled embodiment). We do not think in abstraction but in and through our bodies, with their sensations, needs, vulnerabilities, and capacities for pleasure and pain. Any bioethical reflection that neglects embodiment risks treating persons as disembodied decision-makers rather than incarnate beings (Merleau-Ponty 1945/2012). To attend to bodily sense means recognizing that embodiment is *experienced*,* interpreted*,* and valued*. Pain is not only nociceptive but lived suffering that shapes one’s sense of existence. Disability is not mere dysfunction but a mode of bodily being-in-the-world that carries its own experiential texture and social meaning (Shakespeare 2013). Vitality, haleness, or *joie de vivre* are not mere physiological facts but embodied senses of aliveness.

Debates over palliative sedation illustrate this concretely. Standard analysis asks whether sedation constitutes killing, violates non-maleficence, or satisfies consent requirements (Cherny and Radbruch 2009; Holland 2016). The bedside situation is more complex: for instance, a patient with refractory existential suffering at the end of life, the family present but uncertain whether to stay at the bedside, the nurse adjusting the infusion, the attending physician balancing symptom relief against the patient’s wish to remain communicatively present for as long as possible. Procedural questions about intent, dosage, and consent can be discharged with reasonable confidence – but does sedation preserve a meaningful embodied presence? For the patient, this is the difference between dying as a continuous self and being reduced to bare life; for clinicians and family, it shapes how the death will be remembered and integrated. Bodily sense expands bioethics from physiological viability to lived embodiment.

*Sense of Self*: If the body is the ground of existence, the self is its narrative centre. The sense of self is anchored primarily in Person (narrative identity), with extensions into Society and Work (recognition, responsibility and role). Human beings do not merely experience; they interpret themselves as agents with a past, present, and future (Ricoeur 1992). Illness, disability, or trauma can profoundly disrupt this narrative and biographical identity, challenging one’s ability to see oneself as the author of a coherent life. A practice of bioethics attentive to *bios* as existential and philosophical health asks not only whether a treatment sustains physiological integrity but whether it supports the patient’s capacity to sustain or reconstruct a meaningful sense of self. This is particularly evident in neurodegenerative diseases such as Alzheimer’s dementia. Clinical ethics tends to focus on questions of consent, capacity, and substitute decision-making (Holland 2016, 58–62). Yet further questions should not be avoided: can one’s sense of self remain strong and meaningful even when memory fades? What does it mean to care for someone whose identity has become blurry?

Recent work in narrative medicine has demonstrated that patients’ sense of self is constituted through storytelling, through the capacity to integrate experiences into coherent biographical narratives (Charon 2006). When illness disrupts this narrative capacity, the person experiences not merely physical decline but existential fragmentation. Medical interventions that restore narrative coherence, even if they do not cure disease, can be profoundly therapeutic in enabling patients to reconnect with their sense of who they are. The sense of self thus expands bioethics beyond procedural autonomy toward existential integrity. It recognizes that a meaningful *bios* requires to a certain extent authoring one’s life with some degree of authenticity, authority and authorship.

*Sense of Belonging*: Human beings are relational creatures, constituted by families, communities, cultures, and ecosystems. The sense of belonging is anchored primarily in Society (embeddedness), with extensions into Person (felt belonging), Earth (belonging to a place and a milieu) and Work (belonging to a craft or discipline). A flourishing life depends on the recognition that one is embedded in webs of reciprocity, comparison, adversity, solidarity, and care (Taylor 1989). Modern bioethics acknowledges this through principles of justice, but it often treats belonging in distributive and utilitarian terms: who has access to resources, how are benefits and burdens allocated. While necessary, distributive justice does not capture the full existential texture of belonging.

In other cases, belonging is conflated with nuclear family ties, but belonging is as much existential as political. To lose belonging is to suffer a form of social death even while biologically alive. This became evident during the COVID-19 pandemic. Debates about vaccine allocation were framed largely in terms of justice and equity (Emanuel et al. 2020). For marginalized communities, the issue was not only distribution but *recognition* and the experience was lived as a form of exclusion from global solidarity (Ortiz-Millán 2025). On the other hand, belonging that is imposed is often experienced as ethically and existentially harmful. Similarly, end-of-life care reveals the centrality of belonging. Patients facing death may express fear of abandonment above fear of pain, of dying alone, of being forgotten, of ceasing to matter (Krikorian and Limonero 2012). Palliative care’s emphasis on family presence or communal ritual acknowledges that a good death is not merely pain-free but relationally embedded.

Bioethical deliberation that takes belonging seriously goes beyond distributive fairness. It asks for instance: how do medical practices, public health policies, and research agendas affect the relational fabric of our forms of life? Do they enhance mutual recognition or reinforce exclusion? Only by engaging belonging as a polysemic existential dimension can bioethics address what is genuinely at stake in public health.

*Sense of the Possible*: A life is never simply what it is; it is also what it might become. The sense of the possible is primarily anchored in Earth (horizon) and Person (imagination); it is the dimension where C, the creative flux of reality (or, for short, *Creal*), enters PEWS most directly. Human beings are oriented toward possibilities, imagining futures, projecting goals, experimenting with alternatives, feeling potential. A flourishing life requires the conviction that one’s existence may unfold into new directions, that creativity and transformation remain available, as well as the opening of the unexpected and the unknown. It also requires the ethical capacity to leave some potential unactualized.

When possibility collapses, life becomes stunted or despairing. Depression, for instance, is characterized by the experience that no meaningful future is available, that existence has become empty of possibles (Ratcliffe 2015). Many medical interventions are justified not because they sustain mere survival but because they *open possibilities*: rehabilitation after injury, prosthetics that restore mobility, psychiatric treatment that rekindles agency. Conversely, some interventions may foreclose possibilities. Genetic interventions that narrow the range of acceptable human variation, health systems that restrict options for existential creativity, or psychiatric diagnoses that pathologize neurodiversity can all diminish the sense of the possible (Shakespeare 2013). A practice of bioethics respectful of possibility and potential would ask: what new forms of life are latent in human diversity, and how can society expand their *compossibility* within eudynamic spaces?

The sense of the possible guards against determinism. It resists reducing life to a fixed script, whether genetic, social, or technological. It affirms the openness of *bios* to become otherwise, the capacity for human freedom and existential creativity beyond mainstream norms.

*Sense of Purpose*: Possibility alone is insufficient; human lives require orientation. The sense of purpose is anchored primarily in Work (enactive worldmaking), with extensions into Person (vocation and biographical orientation), Society (collective projects), and Earth (care for the more-than-human). Human beings tend to flourish when engaged in meaningful projects, disciplined vocations, and long-term commitments. A greater purpose anchors existence, giving it self-transcendence, direction and hopeful coherence (Frankfurt 1988). To live without a meaningful purpose is to drift, with the risk of experiencing one’s life as futile, mere survival.

Medicine encounters this dimension constantly. Patients facing serious illness often ask: “What should I live for?” Palliative care recognizes this when it seeks not only to manage pain but to help patients articulate life priorities (Krikorian and Limonero 2012). Standard bioethics tends to treat questions of deep orientation in life as beyond its scope. To integrate a sense of purpose is to acknowledge that bioethical reflection often engages implicit teleological questions: what existential end does this intervention serve? Does it enable the patient to pursue projects that matter to them? Does it sustain their ability to contribute, to care, to create? As Viktor Frankl argued from his experience in concentration camps, humans can endure immense suffering if they perceive meaning and a greater purpose; they collapse and may become hypersensitive when existence is meaningless (Frankl 1946/2006).

Sense of purpose thus reframes bioethics from managing survival to enabling meaningful sustainable agency. It recognizes that what matters is not merely how long one lives but toward what self-transcendent ideals one’s life is directed.

*Philosophical Sense*: Finally, the philosophical sense integrates the others. It is co-anchored in Earth (cosmos and the more-than-human horizon), Society (worldview as embedded cosmology), and operates integratively across all four PEWS domains in articulating the relation between PEWS and C. Humans situate their lives within explicit or implicit worldviews (religious, humanist, naturalist, ecological, utilitarian, etc.) that frame suffering, mortality, and care. Even claims of neutrality presuppose a worldview. A practice of bioethics attentive to this dimension does not impose a doctrine but recognises that ultimate questions are often implicitly present in experiences of health and illness.

This philosophical dimension is often ignored in secular bioethics, which may seek a form of neutrality in matters of ideology or worldview. But even the attempt to remain neutral can become itself a worldview or an implicit ideology. Patients and communities tend to interpret illness, suffering, and death through cosmological frames pertaining to structural meaning. To neglect this is to treat persons reductively.

A bioethical framework attentive to our philosophical sense would not impose a single worldview but would create space for reflection, dialogue, and plural interpretation. It would recognize that ultimate questions and systematisations are implicitly present in the experience of health, illness, and care. More importantly, it would treat philosophical reflection itself as a form of care: a way of integrating the dimensions of life into a relieving practice of meaning.

Taken together, these six dimensions (bodily sense, sense of self, sense of belonging, sense of the possible, sense of purpose, philosophical sense) articulate the structure of *bios* as an integrated ecology of sense-making. A life flourishes when these dimensions cohere: when bodily vitality supports narrative coherence, when relational belonging opens imaginative possibilities, when purposive projects are grounded in cosmological orientation.

This integration may be captured in a heuristic formula: T = PEWS + C: Thinking (eudynamic cognition) emerges from the integration of Person (embodied cognition and biography), Earth (extended cognition, both instrumental and natural), Work (enactive skilled transformation), and Society (embedded social cognition), plus Creal, the creative unknown that exceeds realized systematization (de Miranda 2025).

By asking how medical practices and policies affect these dimensions, bioethics can move beyond the management of *zoē* to the cultivation of *bios*. In the next section, I indicate what this means for clinical practice, institutional policy, and bioethics education.

## Implications for practice, policy, and pedagogy

The framework of Eudynamic Health promises practical orientations for how bioethical reflection may be extended in clinical settings, institutional contexts, and educational programs. To say that bioethics is about cultivating *bios ethikos* is to engage in a reconfiguration of practice. In what follows, I consider three domains (clinical ethics, institutional policy, and pedagogy) and indicate how eudynamic bioethics might transform them.

Clinical ethics consultations are often bioethics’ most visible intervention. Consultants are summoned to resolve disputes, mediate between families and clinicians, or interpret complex cases. Their interventions are more often than not framed within procedural paradigms: balancing respect for autonomy and beneficence, clarifying informed consent, ensuring compliance with law and policy (Holland 2016, 45–67). While these tasks are necessary in principle, they risk neglecting the existential dimensions of the patient’s life. Bioethics rooted in Philosophical Health would clearly recentre ethics consultations as a practice of sense-making across at least the aforementioned six dimensions. When a patient faces a critical decision, for instance whether to continue life-sustaining treatment, whether to accept risky surgery, or whether to enrol in experimental therapy, the central issue, beyond medical risk or informed consent, is the *meaning* of this choice within the patient’s philosophy of life, and how, beyond the personal concern, this meaning is articulated within PEWS + C.

A terminally ill patient refusing further chemotherapy is today likely to be confronted to a proceduralist approach asking whether the patient has decision-making capacity, whether they have received adequate information about risks and benefits, and whether their refusal complies with legal and institutional standards (Beauchamp and Childress 2019). These questions are indispensable, and in several cases sufficient to justify a course of action. However, even when they are answered, they rarely exhaust what is ethically at stake. What remains unresolved is not a procedural failure, but how this decision resonates across the broader conditions of the patient’s life and its compossible entanglement with others. From a eudynamic bioethical perspective, such questions are not peripheral but should become central. Ethical deliberation that remains confined to autonomy and consent risks overlooking how decisions function as acts of existential valuation and interpretation rather than mere preference expression.

In operational terms, this contributes to shared decision-making not by adding a new procedural step to clinical encounters but by cultivating a particular quality of listening that informs the dialogue between clinicians, patients, and families. A PEWS-informed clinician or ethics consultant – a *eudynamicist*, that is, a practitioner of eudynamic care – holds in mind a small set of orienting questions, distributed across the four dimensions: at the level of *Person*, what is the patient trying to preserve or to relinquish in their own embodied and biographical sense of who they are? At the level of *Earth*, how does this decision relate to the wider milieu and material conditions within which the patient is situated? At the level of *Work*, what does meaningful activity, vocation, or enactive contribution look like under the patient’s altered conditions? At the level of *Society*, what is the texture of the patient’s relational fabric, and how do collective expectations shape what can be voiced? These questions function as a discreet attentional grid that allows the clinician to register dimensions of meaning that strictly procedural protocols neglect to articulate, and to invite the patient to speak from those dimensions when relevant: body, self, belonging, possibility, purpose, philosophical sense.

When thought holistically through PEWS + C, a decision unfolds across multiple dimensions. At the level of Work, clinicians and caregivers confront tensions between professional roles oriented toward life-prolongation and the recognition that continued intervention may no longer constitute meaningful care. Moral distress in such contexts does not necessarily signal ethical error; it may be understood as the existential remainder produced by professional practice: procedural justification succeeds, while the lived meaning of caregiving becomes strained (Jameton 1984; Epstein and Hamric 2009).

At the level of Society, end-of-life attitudes are shaped by cultural narratives that valorise endurance, resilience, and “fighting” disease, as well as by institutional expectations concerning responsible use of resources. Such decisions may implicitly reinforce social imaginaries that equate ethical success with survival at all costs, while also revealing how certain forms of dying are perceived as more intelligible or legitimate than others (Pellegrino 1992; Ricoeur 1992). These societal dimensions tend to remain backgrounded in individual ethics consultations, yet they decisively shape the collective horizon within which choices appear meaningful.

The Earth dimension is implicated in a more material sense. Chemotherapy is embedded in technoscientific infrastructures (pharmaceutical production, hospital systems, energy-intensive care environments, regional disparities, etc.) that condition what forms of life are sustained and how. Refusing treatment may therefore be read as a withdrawal from a particular technological mode of existence. Even if such considerations cannot be easily resolved at the bedside, their ethical relevance can be progressively acknowledged as part of the wider material and ecological configuration within which decisions are made (Potter 1971; Emanuel et al. 2020).

Across these dimensions, what emerges is the existential remainder: the residue of ethical significance that persists once a standard version of bioethics has fulfilled its justificatory function. This remainder does not indicate a failure of ethics, but a boundary of institutional rationality. Ethical responsibility is necessarily distributed across roles, levels, and temporal horizons. Eudynamic bioethics does not pretend to integrate all dimensions at once, here and now, but to render these distributions visible and ethically intelligible or even actionable in the longer term.

At the level of institutional policy, this perspective invites reflection beyond procedural compliance. Research review boards, triage protocols, and hospital guidelines may succeed in minimising risk and ensuring fairness while still generating existential remainders at the level of professional identity, public trust, or collective orientation. A PEWS-informed analysis does not replace survival-based criteria or regulatory safeguards, but it situates them within a broader ethical horizon by asking how policies shape the conditions under which lives and practices remain inhabitable over time (Pellegrino 1992; Sen 1999). Institutions already make implicit judgments about what kinds of lives matter; making these judgments explicit allows for democratic deliberation and accountability.

Finally, bioethics education must attend to these dynamics. Curricula centred exclusively on principlism, case analysis, and regulatory knowledge may risk producing practitioners primarily trained in procedural justification and leave practitioners ill-prepared to recognise when ethical significance has been displaced rather than resolved with a concern for the existential and philosophical health perspective. A pedagogical approach informed by the latter complements analytical rigor with capacities for narrative interpretation, institutional reflexivity, sense-making dialogue, and boundary awareness. In this spirit, there is today a growing worldwide net of counsellors trained in sense-making interviews looking at elements of philosophical health (*SMILE_PH*) via the Philosophical Health International network. Students learn how to identify via phenomenological semi-structured dialogue how existential remainders are distributed across persons, practices, and institutions (de Miranda 2024).

By making visible how ethical success at one level may generate remainders at another, eudynamic bioethics allows bioethics to reflect on its own boundaries without abandoning its practical vocation. Attending to the existential and intercreative remainder situates ethical thinking within a more adequate understanding of what ethical decisions do in lived, institutional, and collective contexts, in the spirit of *bios ethikos* we have examined in this article.

## Conclusion

Bioethics has become an indispensable component of modern institutional life. It regulates intervention, secures consent, distributes scarce resources, and provides justificatory frameworks for decision-making under conditions of technological and moral complexity. The argument of this article has not been that bioethics should retreat from its institutional vocation. Rather, it has been that institutional bioethics regularly encounters a boundary: the point at which procedural success leaves ethically significant dimensions of lived existence only partially articulated.

Ethical thinking (T) can be institutionally structured through systematic attention to the distributed conditions of human existence: Person, Earth, Work, and Society (PEWS). These four dimensions, grounded in a phenomenologically informed understanding of distributed cognition, allow for ethical evaluation to move beyond binary dilemmas toward plural configurations, what might be called four-dimensional or *tetralemmatic* reasoning (rather than dilemmatic). In it, decisions are traced across embodied experience, ecotechnological extension, skilled practice, and social or cultural embeddedness.

Importantly, PEWS is not merely descriptive; it can also inform operational reflection. Institutional bioethics already employs structured evaluative tools (risk–benefit matrices, quality-adjusted life years, procedural checklists, etc.). A eudynamic extension might not replace such instruments but complement them with multidimensional assessments capable of tracking existential and relational effects alongside clinical outcomes. The Philosophical Health Compass (PHC), with its 48 stances on a Likert scale (de Miranda et al. 2025), offers one instance of such operational application. Used appropriately, such instruments can render aspects of existential health partially measurable while preserving qualitative depth if they are associated with sense-making dialogue.

The formula T=PEWS + C does not end with a systematization. The addition of C for the Creal or Creative Real marks a decisive qualification. If PEWS designates what can be articulated, distributed, and institutionally structured, C designates what remains open: the emergence of new meanings, reorientations, and creative or unexpected events that exceed prior categories. No evaluative instrument, however sophisticated, can exhaust the horizon of possibility. To attempt a full systematisation of care would be to risk transforming bioethics into a totalising apparatus of existential governance.

The tension between PEWS and C therefore defines the ethical posture of eudynamic bioethics. On the one hand, institutions should not disavow responsibility for the existential consequences of the practices they shape. On the other hand, they must remain vigilant against overreach. Institutionalising PEWS thinking enables a more adequate evaluation of how decisions affect the distributed conditions of existence. But maintaining awareness of C as universal creative onflow preserves humility before the generative indeterminacy and the ambivalence of human experience.

Eudynamic bioethics seeks to articulate what can be more integratively structured while preserving openness to what exceeds systematisation. PEWS enables a multidimensional evaluation of how decisions affect embodied, ecological, professional, and social conditions of life. The addition of C for the openness of existential possibility guards against a totalising form of systematisation. The return to *bios ethikos* asks whether the governance of life sustains the conditions under which existences remain diversely meaningful and open to further creative becoming.

## References

[CR1] Ackrill, J.L. 1980. Aristotle on Eudaimonia. In *Essays on Aristotle’s Ethics*, ed. Amélie Oksenberg. Rorty, 15–33. Berkeley: University of California Press.

[CR2] Agamben, Giorgio. 1998. *Homo Sacer: Sovereign Power and Bare Life*. Translated by Daniel Heller-Roazen. Stanford, CA: Stanford University Press.

[CR3] Agamben, Giorgio. 2016. *The Use of Bodies*. Translated by Adam Kotsko. Stanford, CA: Stanford University Press.

[CR4] Arendt, Hannah. 1958. *The Human Condition*. Chicago: University of Chicago Press.

[CR5] Aristotle. 1999. *Nicomachean Ethics*. Translated by Terence Irwin. 2nd ed. Indianapolis, IN: Hackett Publishing.

[CR6] Beauchamp, Tom L., and James F. Childress. 2019. *Principles of Biomedical Ethics*. 8th ed. New York: Oxford University Press.

[CR7] Charon, Rita. 2006. *Narrative Medicine: Honoring the Stories of Illness*. Oxford: Oxford University Press.

[CR8] Cherny, Nathan I., and Lukas Radbruch. 2009. European Association for Palliative Care (EAPC) recommended framework for the use of sedation in palliative care. *Palliative Medicine* 23 (7): 581–93. 10.1177/0269216309107024 19858355

[CR9] Clark, Andy, and David J.. Chalmers. 1998. The extended mind. *Analysis* 58 (1): 7–19.

[CR10] de Miranda, Luis, Charlotta Ingvolstad Malmgren, Jonathan E. Carroll, Caroline S.. Gould, Rodney King, Christian Funke, and Sena Arslan. 2025. The philosophical health compass: A new and comprehensive assessment tool for researching existential dimensions of wellbeing. *Methodological Innovations* 18 (3): 129–142.

[CR11] de Miranda, Luis. 2021. Think into the place of the other: The Crealectic approach to philosophical health and care. *International Journal of Philosophical Practice* 7 (1): 89–103.

[CR12] de Miranda, Luis. 2024. *Philosophical Health: A Practical Introduction*. London: Bloomsbury.

[CR13] de Miranda, Luis. 2025. Crealectics as a Creative Method: A New Approach to Holistic Innovation for Compossible Worlds. Cham: Palgrave Macm illan

[CR14] Emanuel, Ezekiel J., Govind Persad, Ross Upshur, Beatriz Thome, Michael Parker, Aaron Glickman, Cathy Zhang, Connor Boyle, Maxwell Smith, and James P. Phillips. 2020. Fair allocation of scarce medical resources in the time of Covid-19. *New England Journal of Medicine* 382 (21): 2049–55. 10.1056/NEJMsb2005114 32202722

[CR15] Epstein, Elizabeth G., and Ann B. Hamric. 2009. Moral distress, moral residue, and the crescendo effect. *Journal of Clinical Ethics* 20 (4): 330–42. 20120853

[CR16] Fassin, Didier. 2009. Another politics of life is possible. *Theory, Culture and Society* 26 (5): 44–60.

[CR17] Foucault, Michel. 1973. *The Birth of the Clinic: An Archaeology of Medical Perception*. Translated by A.M. Sheridan Smith. New York: Vintage Books.

[CR18] Frankfurt, Harry G. 1988. *The Importance of What We Care About: Philosophical Essays*. Cambridge: Cambridge University Press.

[CR19] Frankl, Viktor E. 2006. *Man’s Search for Meaning*. Boston: Beacon Press. [1946].

[CR20] Gallagher, S. 2021. *Embodied and enactive approaches to cognition*. Cambridge: Cambridge University Press.

[CR21] Gallagher, Shaun, and Dan Zahavi. 2008. *The Phenomenological Mind: An Introduction to Philosophy of Mind and Cognitive Science*. London: Routledge.

[CR22] Gallagher, Shaun. 2013. The socially extended mind. *Cognitive Systems Research* 25–26: 4–12.

[CR23] Hadot, Pierre. 1995. *Philosophy as a Way of Life: Spiritual Exercises from Socrates to Foucault*. Edited by Arnold I. Davidson. Translated by Michael Chase. Oxford: Blackwell Publishing.

[CR24] Holland, Stephen. 2016. *Introducing Bioethics*. Cambridge: Polity Press.

[CR25] Husserl, Edmund. 1970 [1936]. *The Crisis of European Sciences and Transcendental Phenomenology*. Translated by David Carr. Evanston, IL: Northwestern University Press.

[CR26] Irwin, Terence. 1999. Aristotle’s Ethics. In *The Cambridge Companion to Aristotle*, edited by Jonathan Barnes, 195–227. Cambridge: Cambridge University Press.

[CR27] Jameton, Andrew. 1984. *Nursing Practice: The Ethical Issues*. Englewood Cliffs, NJ: Prentice-Hall.

[CR28] Jaspers, Karl. 1959. *Philosophy*. Vol. 2. Translated by E.B. Ashton. Chicago: University of Chicago Press.

[CR29] Jaspers, Karl. 1997 [1913]. *General Psychopathology*. Translated by J. Hoenig and Marian W. Hamilton. Baltimore: Johns Hopkins University Press.

[CR30] Jonsen, Albert R. 1998. *The Birth of Bioethics*. New York: Oxford University Press.

[CR31] Krikorian, Alicia, and Joaquín T. Limonero. 2012. An integrated view of suffering in palliative care. *Journal of Palliative Care* 28 (1): 41–9. 22582471

[CR32] Lemke, Thomas. 2011. *Biopolitics: An Advanced Introduction*. New York: New York University Press.

[CR33] Merleau-Ponty, Maurice. 2012 [1945]. *Phenomenology of Perception*. Translated by Donald A. Landes. London: Routledge.

[CR34] Mills, Catherine. 2018. *Biopolitics*. London: Routledge.

[CR35] Ortiz-Millán, Gustavo. 2025. Justification and limitations of the duty to treat. *The Journal of Medicine and Philosophy* 50 (5): 354–69. 10.1093/jmp/jhaf017 PMC12534082 40366910

[CR36] Pellegrino, Edmund D. 1992. The metamorphosis of medical ethics: A 30-year retrospective. *Journal of the American Medical Association* 269 (9): 1158–62. 8433472

[CR37] Plessner, Helmuth. 2019 [1928]. *Levels of Organic Life and the Human: An Introduction to Philosophical Anthropology*. Translated by Millay Hyatt. New York: Fordham University Press.

[CR38] Potter, Van Rensselaer. 1971. *Bioethics: Bridge to the Future*. Englewood Cliffs, NJ: Prentice-Hall.

[CR39] Ratcliffe, Matthew. 2015. *Experiences of Depression: A Study in Phenomenology*. Oxford: Oxford University Press.

[CR40] Ricoeur, Paul. 1992. *Oneself as Another*. Translated by Kathleen Blamey. Chicago: University of Chicago Press.

[CR41] Robbins, Philip, and Murat Aydede, eds. 2009. *The Cambridge Handbook of Situated Cognition*. Cambridge: Cambridge University Press.

[CR42] Sen, Amartya. 1999. *Development as Freedom*. New York: Alfred A. Knopf.

[CR43] Shakespeare, Tom. 2013. *Disability Rights and Wrongs Revisited*. 2nd ed. Abingdon: Routledge.

[CR44] Shapiro, Lawrence. ed. 2014. *The Routledge Handbook of Embodied Cognition*. London: Routledge.

[CR45] Taylor, Charles. 1989. *Sources of the Self: The Making of the Modern Identity*. Cambridge, MA: Harvard University Press.

[CR46] Thompson, Evan. 2007. *Mind in Life: Biology, Phenomenology, and the Sciences of Mind*. Cambridge, MA: Harvard University Press.

[CR47] Varela, Francisco J., Evan Thompson, and Eleanor Rosch. 1991. *The Embodied Mind: Cognitive Science and Human Experience*. Cambridge, MA: MIT Press.

[CR48] Williams, Bernard. 1985. *Ethics and the Limits of Philosophy*. Cambridge, MA: Harvard University Press.

